# Thermal Degradation and Chemical Analysis of Flame-Retardant-Treated Jute Fabrics

**DOI:** 10.3390/polym16142049

**Published:** 2024-07-18

**Authors:** Most. Setara Begum, Michael Hummel, Sumit Mandal, Shahriare Mahmood, Md. Reazuddin Repon, Rimvydas Milašius

**Affiliations:** 1 Faculty of Mechanical Engineering and Design, Kaunas University of Technology, Studentu Str. 56, LT-51424 Kaunas, Lithuania; reazmbstu.te@gmail.com (M.R.R.); rimvydas.milasius@ktu.lt (R.M.); 2 Department of Bioproducts and Biosystems, Aalto University, 02150 Espoo, Finland; michael.hummel@aalto.fi; 3 Department of Design and Merchandising, Oklahoma State University, Stillwater, OK 74078, USA; sumit.mandal@okstate.edu; 4 Industrial Engineering and Management, University of Oulu, 90014 Oulu, Finland; shahriare.mahmood@spinnova.fi; 5 Department of Textile Engineering, Daffodil International University, Dhaka 1216, Bangladesh

**Keywords:** jute, flame-retardance, TGA, residual mass, free formaldehyde, ITOFLAM CPN

## Abstract

Jute is an inherent lignocellulosic fiber, consisting of hemicellulose, α-cellulose, and lignin. Industrial ventilation, automotive composites, upholstery, carpets, military uniforms, hospital furnishings, and curtains necessitate the integration of flame-retardance properties into jute fibers. In this investigation, seven weave-structured jute fabrics were treated using an organophosphorus-based flame-retardant (FR) chemical (ITOFLAM CPN) and a crosslinking agent (KNITTEX CHN) by the pad–dry–cure method. The thermal stability, degradation and pyrolysis behavior of jute was measured using a thermogravimetric analyzer (TGA). Surface morphology and element distribution were scrutinized utilizing a scanning electron microscope (SEM) and an energy-dispersive spectrometer (EDS). The ATR-FTIR (Attenuated Total Reflection-Fourier Transform Infrared Spectrometer) technique has been employed for analyzing the composition of chemicals in the jute fabrics. According to the protocols specified in ISO 14184-1, free formaldehyde detection was carried out on the jute fabrics. The flame-retardance property was significantly improved on all of the jute fabrics after FR treatment. FTIR and SEM-EDS studies revealed the presence of FR chemical deposition on the surface of the jute fabrics. TGA analysis indicated that the fabrics treated with FR exhibited premature degradation, leading to the generation of more char compared to untreated samples. The jute fabrics specifically demonstrated a notable enhancement in residual mass, exceeding 50% after FR treatment. However, it is noteworthy that the FR-treated fabrics exhibited an elevated level of free formaldehyde content, surpassing the permissible limit of formaldehyde in textiles intended for direct skin contact. The residual mass loss percentage after ten washes of FR-treated fabrics remained in a range from 32% to 36%. Twill weave designed fabrics (FRD4 and FRD5) clearly showed a lower thermal degradation temperature than the other weaves used in this study.

## 1. Introduction

The structure and chemical compositions of cellulosic and ligno-cellulosic fibers have a significant impact on their thermal behavior. Jute is a plant-based fiber characterized by its composition, which consists of hemicellulose (24%), α-cellulose (60%), and lignin (14%). Due to chemical variations, the thermal behaviors of various components should differ [1]. Earlier investigations [2,3] delved into preliminary research on thermal properties and FR coatings. In order to mitigate the occurrence of fatalities and injuries resulting from fires, efforts have been made to impart fire resistance to jute carpet backing, decorative furnishing fabrics made of jute, and brattice cloth for mines. FR jute fabrics are presently utilized in a range of products, encompassing industrial ventilation, military uniforms, carpets, mats, floor coverings, hospital furnishings, curtains for medical facilities, and various other applications [4]. The principal obstacles related to treating fabrics based on jute for fire protection include elevated chemical add-on, a discernible reduction in tensile strength, and the occurrence of yellowing. Moreover, a majority of these FR formulations require substantial doses of necessary chemicals and are either not durable or only semi-durable [5]. An essential quality of textile materials is flame-retardancy, which helps protect wearers from dangerous clothing. Floor coverings, furniture, and curtains must be flame-resistant when used in public facilities, especially for firefighters and emergency personnel. There are numerous requirements for fire retardancy in the aviation and military sectors as well [6,7].

For decades, there have been attempts to address the risks posed by combustible materials. Alum, ferrous sulphate, and borax were combined to create an FR compound that was patented by the English scientist Jonathan Wyld in 1735 [8]. Gay Lussac, a distinguished chemist, innovatively developed an FR finish specifically designed for linen and hemp fabrics in 1821. This groundbreaking approach involved utilizing different ammonium salts, either including or excluding borax, representing the initial systematic effort to impart flame resistance to textiles [9]. The chemistry of textile substrates is quite varied, and the flame-retardancy realm is broad and intricate. FR chemicals and formulations encompass a diverse array of forms and combinations of halogen, phosphorus, nitrogen, sulfur, boron, and antimony, as well as various other elements. Applying chemicals to 10% to 30% of the weight of the materials is necessary for FR treatments. Hence, the aesthetic properties, physical and tensile properties, as well as creasing and pilling capabilities, might exhibit differences. FR cotton and synthetic fibers like Kevlar, Nomex, and PBI provide protection to the wearer due to their non-shrinking properties in the presence of flames. Nevertheless, thermoplastic fibers successfully undergo the ignition or flammability test by retracting away from the fire; however, in case of practical situations, individuals wearing the attire encounter direct exposure to heat and hence are burnt by the molten mass that comes in contact with the body [7].

Currently, one of the major challenges that researchers are facing is the durability of the flame retardancy of jute-based products. A variety of FR formulations have been tested on jute materials for use in mines, automobile upholstery, and household textiles, especially for cooking aprons and gloves, drapes, and curtains in public spaces because these materials are prone to flame. Various durable and non-durable FRs can be applied to impart flame-resistance to cellulosic materials for diverse applications. Nonetheless, standardizing the FR finish recipe and application method is important for ligno-celluloses, especially for jute and jute-blended textiles [10]. Generally, jute and other cellulosic fibers are highly flammable materials, and the main difference from cotton is that jute contains a significant amount of lignin and hemicellulose in its chemical constituents [11].

There is limited research on natural fibers beyond cotton. Nevertheless, a study on hemp, wool, silk, and linen was examined by Yusuf [12], and the flame-retardance of jute was explored by Mehta and Hoque [13]. Dorez et al. examined the influence of hemicellulose, cellulose, and lignin on the thermal decomposition and burning behavior of natural fibers [14]. In earlier investigations, jute was treated with various organophosphates as well as with other chemicals, such as sodium metasilicate nonahydrate, diammonium hydrogen phosphate, ortho-phosphoric acid, urea, borax, ammonium sulfamate, thiourea, etc. [1,15,16,17,18,19,20]. In a more contemporary investigation, Samanta et al. explored the flame-retardance finish treatment on jute fabric by incorporating nano-zinc oxide [21] and Li et al. studied the flame-retardance of jute by using chitosan and sodium alginate [22]. Roy et al. also noted sustained FR properties in jute [11].

Although previous researchers have investigated the flame-retardancy of jute fabrics, it is notable that most of the research has focused on the flame-retardant finish used on the fabrics. None of this research has focused on the impact of different jute fabric structures on the flame-retardant coating and overall flame-retardancy performance of the fabrics. The novelty of this study is how the different fabric designs could have an impact on jute fabrics with this FR formulation. This study is important in order to know the impact of weave structure on the flame-retardancy of jute fabrics, even though they have been coated with the same FR finish. This will advance the field of polymer and textile science by developing knowledge on flame-retardancy through the structure of the fabrics. In the future, researchers could develop this structure to obtain better flame-retardancy instead of using FR chemicals; this could be a sustainable approach to imparting flame-retardancy to fabrics.

In this study, FR treatment (ITOFLAM CPN) was employed along with a crosslinking agent (KNITTEX CHN) on seven different weave designed pure jute fabrics, and the flame-retardance performance was investigated. These fabrics were investigated through an extensive analysis to conclude our findings as per the need of the scientific field. For an extensive analysis, the FR-treated fabrics were further evaluated by thermal degradation analysis, FTIR, and SEM-EDS studies. This study extends the FR analysis to seven weave structures of jute fabrics and evaluates the influence of weave on the flame-retardance performance among them, as no such studies are reported at present.

## 2. Materials and Methods

### 2.1. Materials

All tests were conducted employing 100% jute with seven different structurally designed woven fabrics. Detailed designations are summarized in Table 1. The jute fabrics were manufactured at Janata Jute Company, Dhaka, Bangladesh.

**Figure 1:**
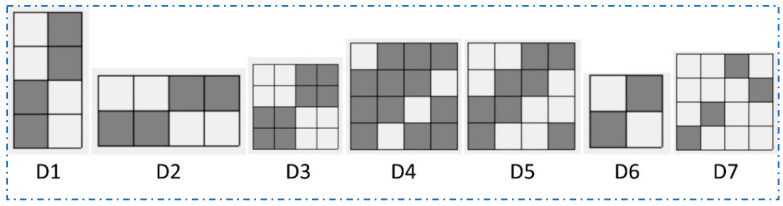
Weave designs of seven different woven jute fabrics.

Table 1 describes the specifications of the jute fabric that was used throughout the experiments in this study. There are seven different weave structures of fabrics used in this study, such as Warp Rib (2/2), Weft Rib (2/2), Basket weave (4/4), Twill (3/1), Twill (2/2), Plain (1/1), and 4 ends Irregular Satin. All fabrics contained the same number of warp (26) and weft (20) threads during weaving and for each set of yarn, the linear density was 210.54 Tex. Throughout this study, these jute fabrics are named D1, D2, D3, D4, D5, D6, and D7 and after FR treatment FRD1, FRD2, FRD3, FRD4, FRD5, FRD6, and FRD7, respectively. The weave structures are presented in Figure 1.

### 2.2. Chemicals and Reagents

A commercially available FR chemical based on organophosphorus, ITOFLAM CPN, a melamine resin (crosslinking agent), KNITTEX CHN, and phosphoric acid were supplied by a textile factory in India. A detergent (SCOUTEX SLC), a sequestering agent (MASQUOIL), and sodium carbonate (Na_2_CO_3_) were supplied by Janata Jute Mills, Bangladesh. All chemicals were employed in their received state.

### 2.3. Fabric Preparation

Before the initiation of FR treatment, all of the fabrics were subjected to traditional de-sizing and scouring processes by using SCOUTEX SLC (Detergent) 1 g/L, MASQUOIL (sequestering agent) 1 g/L, and Na_2_CO_3_ 2 g/L at 90 °C for 40 min; the process was carried out for 4 cycles on a Jigger machine. The fabrics were then neutralized with acetic acid 0.5 g/L at 50 °C for 20 min for 2 cycles, and then dried at 110 °C in the flat-bed dryer.

### 2.4. Treatment for FR Finishing

The jute fabrics underwent treatment with ITOFLAM CPN along with a crosslinking agent (KNITTEX CHN) using the pad–dry–cure (2-dip-2-nip) method followed by a laboratory-scale vertical padder. The FR treatment recipe and parameters are described in Table 2. ITOFLAM CPN is an organophosphorus-based FR chemical used in this work. However, to complete the reaction between cellulose and this FR chemical, one acidic catalyst is needed along with heating/curing. In this case, a phosphoric acid catalyst was used for its better compatibility with the above-mentioned FR chemical. Subsequently, the samples were dried at 120 °C for 1 min and cured at 170 °C for 2 min on a laboratory-scale stenter machine (Mathis, CH-8156, Oberhasli, Switzerland). Thereafter, the FR-treated jute samples were thoroughly washed with 0.15 g/L AATCC 193 standard detergent for 5 min. The liquor ratio of the fabrics to the washing solution was 1:50. The solution temperature was 40 °C. Subsequently, the samples were removed, rinsed with deionized water, and then dried at 70 °C for 30 min.

### 2.5. Characterization of Fabric Surface

SEM combined with EDS (ZEISS, Sigma VP, Jena, Germany) was utilized to analyze the surface topography and perform elemental analysis on the jute fabric samples. The best results of the flammability test were achieved on FRD5 fabrics. Therefore, D5 and FRD5 fabrics were selected to show representative results for the analysis of surface topography in this investigation. EDS map analysis was utilized in this study, and the presented data are the average of 3 test measurements. Before scanning, the fabric samples were coated with gold. The alterations in chemical composition induced by the FR treatment were investigated in both untreated and FR-treated jute fabrics, utilizing ATR-FTIR (PerkinElmer Spectrum Two FT-IR Spectrometer, Waltham, MA, USA). In this ATR, a beam of infrared light was passed through a diamond crystal plate in such a way that it reflected at least once off the internal surface in contact with the jute fabrics and scanned the fabrics to observe their chemical compositions. The samples were conditioned for 24 h prior to FTIR tests. Since the ATR mode was utilized for the FTIR analysis, solid fabrics were directly used for measurements. The range of analysis was 4000–500 cm^−1^, with 4 cm^−1^ resolution and scan number 16 in order to obtain a stable, repeatable, reproducible, and noise-free scan of the fabrics. In this study, the fraction of incident infrared radiation that passed through the fabrics in FTIR was used to characterize the interaction of infrared radiation with the fabrics. As a result, the transmittance through the fabrics was mathematically calculated by the ratio of the intensity of transmitted light to incident light, and it was expressed as percentages. A higher transmittance% indicates less absorption by the fabrics.

### 2.6. Analysis of Thermal Degradation Properties

Thermogravimetric analysis (TGA) was carried out in a nitrogen atmosphere using a NETZSCH (Jupiter STA 449 F3, Selb, Germany) thermogravimetric analyzer instrument. A 10 mg sample underwent heating from room temperature to 600 °C at a rate of 10 °C/min. To remove excess moisture from the jute fabrics, all of the jute samples were dried at 90 °C for 20 min prior to TGA analysis. The test was repeated 3 times, and the error bars were adopted based on the std error calculated from the standard deviation of the mean values.

### 2.7. Color Change Measurement Study

Color change measurements were evaluated for untreated and FR-treated jute fabrics utilizing a spectrophotometer (Datacolor SF 800, Lawrenceville, NJ, USA).

### 2.8. Durability Test

To determine the washing durability of the FR performance, the FR-treated samples were washed 5, 7, and 10 times. Samples were washed with 1 g/L commercial detergent for 10 min in a beaker at 40 °C and dried at room temperature. The residual mass of the washed jute samples was investigated by a TGA study followed by the same TGA conditions and parameters stated in Section 2.6. The test was repeated 3 times for each sample.

### 2.9. Free Formaldehyde Content Determination

The quantification of the free formaldehyde content in the jute fabrics adhered to the ISO 14184-1 standards [23] and was assessed utilizing a UV–VIS Spectrophotometer (Lambda 25, Perkin Elmer, Beaconsfield, UK). The absorbances were measured in a 10 mm absorption cell at a wavelength of 412 nm. Equation (1) was employed to compute the quantity of free formaldehyde extracted for each sample (*wF*) in ppm. The test was repeated 3 times for each sample. The error bars were adopted based on the std error calculated from the standard deviation of mean values.

(1) $$wF=\frac{\rho *100}{m}$$

In this equation, the concentration of free formaldehyde (HCHO) extracted for each specimen (*wF*) in ppm, ρ stands for the concentration of HCHO in the solution, measured in mg/L as determined from the calibration graph, while m represents the mass of the specimen, measured in grams.

## 3. Results and Discussions

### 3.1. Assessment of Color Variation

The evaluation of the color variation between the untreated fabrics and those treated with FR was performed by measuring the color difference (ΔE) values of the samples. The results are shown in Table 3. It was assumed that after FR treatment there might have been some color changes in the samples. Following that, the results in this table clearly show some visible color difference values such as 6.61, 6.28, 6.28, 4.24, 3.92, 5.35, and 5.90 for the D1/FRD1, D2/FRD2, D3/FRD3, D4/FRD4, D5/FRD5, D6/FRD6, and D7/FRD7 fabrics, respectively. The maximum color difference was observed on the D1/FRD1 pair, while the minimum was observed on D5/FRD5. Although there is no significant impact of color difference on these fabrics, it may cause some impact if the same treatment is applied on the colored jute fabrics. Moreover, the color change may also be generated due to the cellulose degradation, which may be caused by acidic FR treatment on jute fabrics. The removal of hemicellulose is performed by NaOH treatment, and lignin is removed by an acidic NaClO_2_ solution [24,25,26]. However, for any chemical treatment of jute, there is a small degree of removal of lignin and hemicellulose, depending on the condition of the treatment and the nature of the chemicals applied. Therefore, in the present treatment with this organophosphorus FR chemical in the presence of phosphoric acid, there is a possibility of the removal of a small amount of lignin and hemicellulose, both with little chance of degradation of the cellulose chain in jute [27,28,29]. This degradation could eventually be responsible for the color changes on the FR-treated jute fabric.

During FR treatment, the chemicals get deposited on the surface of the fabric, as well as on the interlacing spaces between the threads in the fabrics; this factor plays a key role in making the fabric surface smooth. Moreover, after the treatment, the threads in the fabric also get closer and are denser, which is clearly visible in the fabric images after the FR treatment. These are ultimately the reasons for making the treated fabric surface smoother than that of the untreated fabrics.

### 3.2. Analysis of Surface Morphology and Chemical Composition

**Figure 2:**
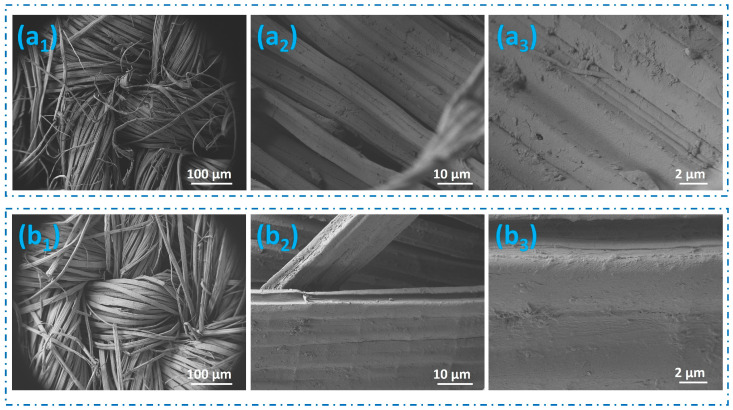
SEM images of (**a_1_**–**a_3_**) untreated jute and (**b_1_**–**b_3_**) FR-treated jute fabrics.

Figure 2 illustrates the surface morphology and uniform dispersion of FR chemicals on jute fabrics (D5 and FRD5) treated with FR, as observed using the SEM. The flame-retardance efficacy of the treated fabrics is affected by how uniformly the FR chemicals are deposited on the fabric surface. Enhanced flame-retardance is often associated with a higher level of chemical consistency on the surface. It can be noted that the untreated samples presented irregular surfaces, whereas the images of jute samples treated with FR displayed more regular and smooth surfaces, signifying that the treatment resulted in the deposition of FR chemicals on the surface of the fiber.

**Figure 3:**
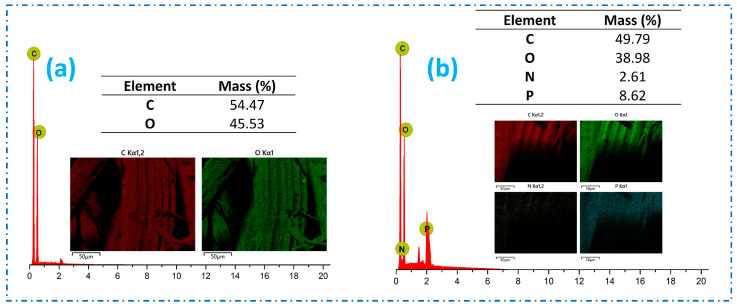
EDS analysis of (**a**) untreated jute and (**b**) FR-treated jute fabrics.

To provide additional illustration, both untreated and FR-treated samples were thoroughly examined and analyzed using energy dispersive spectroscopy (EDS), as depicted in Figure 3. Based on the findings, the untreated sample primarily consisted of elements such as C and O, whereas the FR-treated samples exhibited the presence of N (2.61%) and P (8.62%). It is noteworthy to mention that the distribution of these elements was consistent, thereby creating a physical barrier and imparting flame-retardance properties to the fabrics.

**Figure 4:**
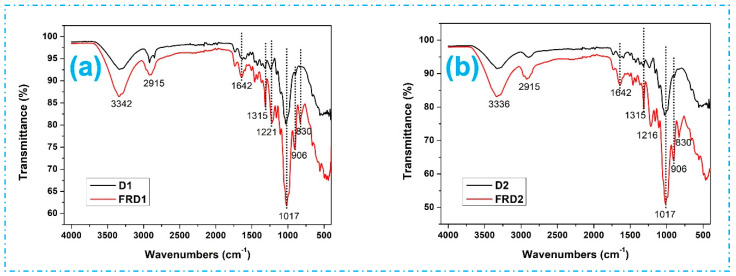

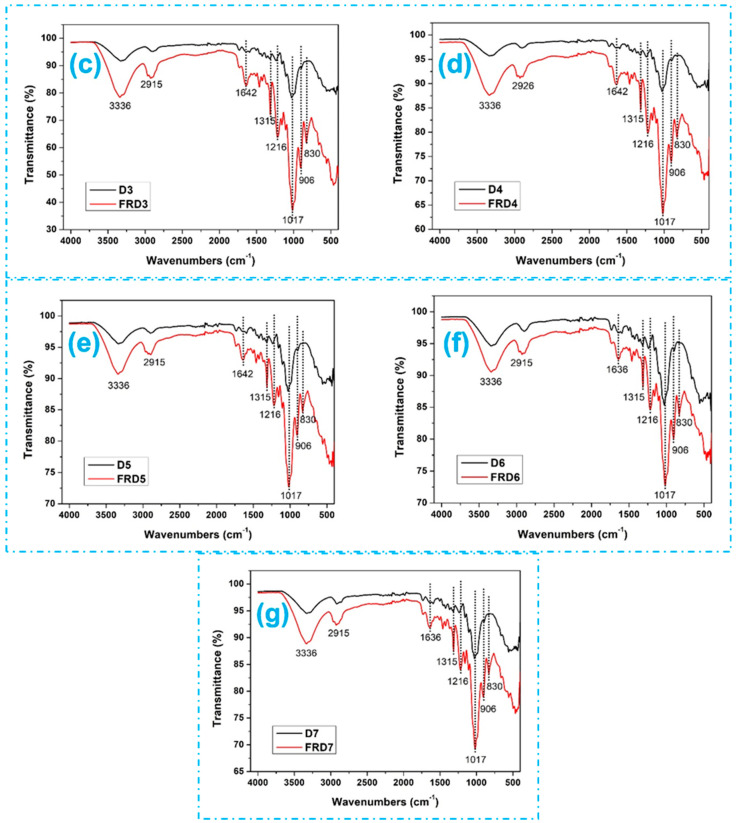
FTIR results of untreated and FR-treated [(**a**) D1 and FRD1; (**b**) D2 and FRD2; (**c**) D3 and FRD3; (**d**) D4 and FRD4; (**e**) D5 and FRD5; (**f**) D6 and FRD6; and (**g**) D7 and FRD7] jute fabrics.

To confirm the chemical reaction between the FR chemical and the jute fibers, the FT-IR spectra of both fabrics were obtained. The ATR-FTIR mode of analysis was employed to ascertain the composition of chemicals on the surface of the substrate. These spectra are focused within the spectral range of 4000 to 500 cm^−1^. As shown in Figure 4, similar FTIR spectra were achieved for all FR-treated fabrics. The peaks observed at 3336 cm^−1^ in the FT-IR spectra of both untreated and FR-treated jute fabrics were associated with the stretching of hydrogen-bonded (OH) groups. This specific band is recognized as one of the identical features of the spectrum related to the α-cellulose in the fiber [30,31]. Likewise, the peaks at 2915 cm^−1^ correspond to the stretching vibrations in C-H bonds of aliphatic methylene groups. This suggests the existence of CH and CH_2_ in cellulose and hemicellulose, as demonstrated by the stretching and bending of C-H bonds [32,33]. The FR-treated spectrum showed an increased peak area at 3336 and 2915 cm^−1^, attributed to increased hydrophilicity, and this demonstrates that the FR-treated fabric has more bound and unbound water molecules that eventually exhibit flame-retardance properties. The prominent peak detected in the FR-treated samples at 1642 cm^−1^ is associated with the H-O-H group of water molecules of the natural fibers [34], and the presence of C=O (carbonyl group) is due to hemicellulose and lignin [35]. The band observed at 1315 cm^−1^ corresponds to the stretching of amide III (C-N) [36,37]. The distinct peak observed at 1017 cm^−1^ was linked to the C-O group found in the hydroxyl and ether groups of cellulose [32,38]. Moreover, the increased peak intensity at 1017 cm^−1^ indicates an augmentation in the -OH and CH-OH wagging of cellulose. This intensification is linked to the stretching vibration of the H_2_O (water) molecules retained by the amide–ester of phosphate compounds present in the applied FR chemical on the jute materials.

The peaks at 906 cm^−1^ were associated with β-glucosidic linkage and indicated the C-H deformation of cellulose through the stretching of C-O-C [39]. Moreover, the peaks at 1216 cm^−1^ and 830 cm^−1^ were designated as the stretching vibrations of P=O and P-C, respectively [40]. These peaks indicate that the jute materials are exposed to phosphorous-based FR chemicals.

### 3.3. Thermal Properties Analysis

**Figure 5:**
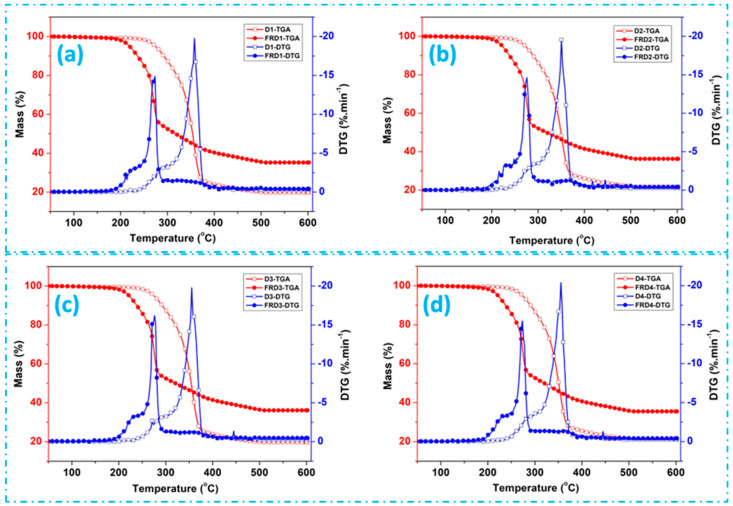

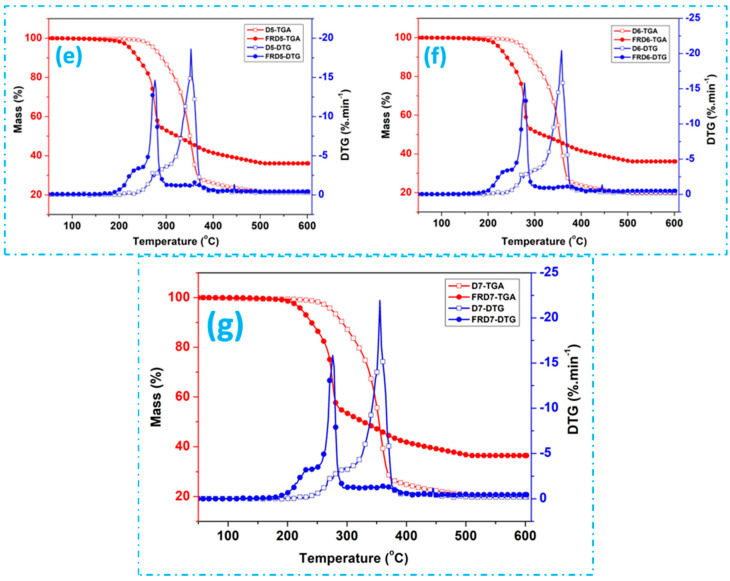
TGA and DTG results of untreated and FR-treated jute fabrics [(**a**) D1 and FRD1; (**b**) D2 and FRD2; (**c**) D3 and FRD3; (**d**) D4 and FRD4; (**e**) D5 and FRD5; (**f**) D6 and FRD6; and (**g**) D7 and FRD7] under nitrogen atmosphere.

TGA and DTG curves for untreated and FR-treated jute fabrics are depicted in Figure 5. The primary pyrolysis stage, characterized by rapid weight loss, occurs at approximately 379 °C for the D1, D2, and D5, and 385 °C, 447 °C, 401 °C, and 390 °C for the D3, D4, D6, and D7 fabrics, respectively. This phase involves dehydration and decarboxylation reactions, resulting in the generation of additional flammable volatiles. The region at approximately 400 °C signifies the breakdown of the char generated in the pyrolysis phase [41]. At lower temperatures around 97–130 °C, a marginal decrease in weight was detected, possibly stemming from the moisture content within the samples. Under typical drying circumstances, it is challenging to reach a totally dry state for hydrophilic polymers. Because of the robust hydrogen bond that forms between hydrophilic groups and water molecules, the dryness of the polymers cannot be precisely determined [42]. It is possible to remove the moisture that is on the surface, but it is rarely possible to break through the drying process of bound water at lower temperatures. Typically, water vaporization stops at temperatures above 100 °C. However, under certain conditions, vaporization can occur at temperatures higher than 100 °C when water molecules are strongly bound by hydrophilic groups [43,44]. Previous studies have reported that the vaporization peak splits into two peaks at around 60 °C and around 120 °C. The high-temperature vaporization peak is associated with the structural alteration of amorphous cellulose chains caused by the desorption of bound water [42]. Past studies have indicated that the primary phase of pyrolysis predominantly involves the degradation of cellulose in the amorphous region of the polymer [45]. A substantial reduction in weight is evident in the jute samples, signifying the pyrolysis of cellulose occurring within the crystalline region of the polymers during the second stage. The predominant results of pyrolysis at this phase comprise the generation of glucose and different varieties of flammable gases [45]. As per previously documented research, the thermal breakdown of cellulose leads to the formation of volatile compounds, both combustible and noncombustible in nature [46]. Continuous reduction in mass loss was observed at elevated temperatures, attributed to processes involving dehydration and decarboxylation, accompanied by the release of water, carbon dioxide, and carbonyl compounds [47].

Generally, for untreated jute, hemicellulose, cellulose, and lignin degrade at around 250–290 °C, 360–365 °C, and at 425 °C, respectively. For the untreated jute fabrics in Figure 5**,** the weight loss rate changes at around 250–270 °C, sharply rising at around 350–370 °C, signifying the degradation of cellulose. Subsequently, it proceeds at a distinct rate until reaching 490 °C, concluding without any additional weight loss.

The slight decrease in weight observed between 425 and 450 or 500 °C, before reaching a point of no additional weight loss at a slower pace, is a result of the decomposition of the lignin component. Early degradation of all three constituents is observed on the FR-treated jute fabrics. This demonstrates that the thermal degradation of the hemicelluloses in FR-treated jute begins and ends between 180 °C and 250 °C. And it continues into the thermal degradation of the cellulose component, initiating between 250 °C and 330 °C at a distinct rapid rate, leading to a faster weight loss but generating more residue. Based on the weight loss data, the degradation of the lignin in FR-treated jute occurs at temperatures of 350–400 °C and beyond. This degradation proceeds at a considerably reduced rate, persisting up to 450 °C and yielding a greater weight of char production, leading to a higher residual weight.

As indicated in Figure 5, a comparable pyrolysis stage is evident for FR-treated jute; however, with a shift of the decomposition peak to a lower temperature at 298 °C for the FRD1 and FRD2 and 308 °C, 292 °C, 302 °C, 328 °C, 329 °C for the FRD3, FRD4, FRD5, FRD6 and FRD7 fabrics, respectively. The initial thermal degradation observed may be attributed to the generation of phosphoric acid while undergoing pyrolysis of phosphorus-containing compounds available in the FR chemical. This results in dehydration, lowering the decomposition temperature and increasing the yield of residual char. Organophosphorus flame retardants help prevent fires by disintegrating in the polymer matrix and producing phosphorus acids that promote char formation and suppress combustion. Furthermore, the structure of phosphorus FRs such as alkyl phosphate, aryl phosphate, phosphonate, and phosphinate significantly influences the mode of action for thermal degradation properties. For all of these compounds, initial degradation occurs because of the elimination of a phosphoric acid based on the level of oxygenation in the phosphorus. In the case of alkyl phosphate, the degradation happens sharply at a low temperature, while it happens at a high temperature for aryl phosphates. However, for phosphonate and phosphinate, degradation occurs slowly at higher temperatures compared to the aforementioned phosphate compounds [48,49].

**Figure 6:**
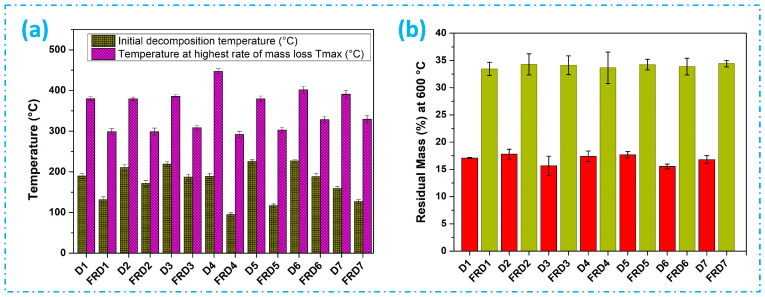
(**a**) Temperature for initial decomposition and at highest mass loss T_max_ (°C) and (**b**) residual mass (%) at 600 °C for untreated and FR-treated jute fabrics.

The temperatures for the commencement of degradation and the highest rate of mass loss, as well as the residual mass at 600 °C, are depicted in Figure 6. These temperatures are extracted from the DTG and TGA curves (Figure 5). Figure 6a evidently shows early decomposition in the FR-treated fabrics compared to the untreated fabrics. The minimum initial decomposition temperature was reported for the FRD4 fabric followed by the FRD5, FRD7, FRD1, FRD2, FRD3, and FRD6 fabrics, respectively. Nonetheless, the temperature at which the highest mass loss was achieved is lower for FR-treated fabrics than for untreated fabrics. The minimum temperature for the final degradation resulted for the D4 fabrics followed by the FRD5, FRD1, FRD2, FRD3, FRD6 and FRD7 fabrics, respectively. Diagram 6(b) illustrates that char residue was more prevalent in jute fabrics treated with FR 33.46%, 34.25%, 34.12% 33.65%, 34.23%, 33.87%, and 34.43% at 600 °C for the FRD1, FRD2, FRD3, FRD4, FRD5, FRD6 and FRD7 fabrics, respectively. On the other hand, the untreated jute fabrics resulted in residual masses of 17.08%, 17.8%, 15.65%, 17.39% 17.67%, 15.53% and 16.8% for the D1, D2, D3, D4. D5, D6, and D7 fabrics, respectively. The residual weights of these samples remain nearly constant above 378 °C for untreated jute and 296 °C for FR-treated jute fabrics. These findings indicate that the FR-treated fabrics formed significantly higher amounts of char compared to the untreated fabrics. This is a unique occurrence as the pyrolysis of cellulosic fabrics was altered due to FR treatment, resulting in cellulosic fibers turning into char due to the initiation of pyrolysis [50]. Previous research has asserted that an augmentation in char production within these temperature ranges aligns with enhanced flame-retardance efficacy [51]. The results of thermal analysis undeniably illustrate a substantial improvement in flame-retardancy in jute fabrics treated with flame retardants [52].

**Figure 7:**
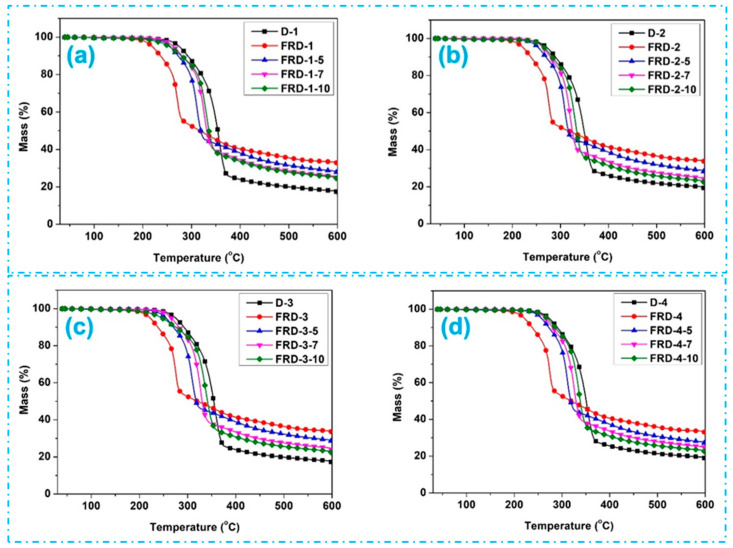

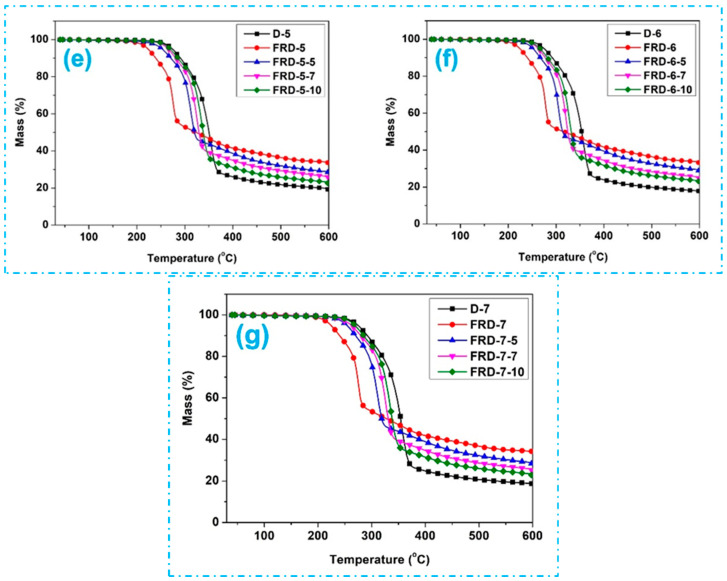
TGA study after different washing cycles of FR-treated jute fabrics: (**a**) FRD1, (**b**) FRD2, (**c**) FRD3, (**d**) FRD4, (**e**) FRD5, (**f**) FRD6, and (**g**) FRD7.

The durability of FR performance was determined by washing the FR-treated jute fabrics for five, seven, and ten washing cycles, and the residual mass percentage was investigated by TGA studies. The results of the durability of washing are presented in Figure 7. The residual mass was reduced after the laundering cycles on all of the jute fabric samples in this study. However, after the maximum number of washing cycles, the fabrics still showed some flame-retardance performance because the residual mass remained higher than their untreated fabric pairs. According to the results presented, after multiple washes, all FR-treated fabrics had some residual mass loss compared to their unwashed conditions. After five washes, the highest mass loss (21.57%) was found in D1 fabrics, while the least mass loss (15%) was found in D6 fabrics. After seven washes, an almost similar mass loss trend is seen, ranging from 26 to 30% in different fabrics. The highest amount of mass loss (36.2%) was after the maximum number of washes (10) in the D3 fabric, while the minimum (32%) was for the D1 fabric. The TGA results after multiple washes of FR-treated jute fabrics indicate that the residual mass remained higher for FR-treated jute fabrics compared to the jute fabrics without any treatment. This is because these jute fabrics are multiphase porous media that have an air phase and solid fiber phase, and the FR chemicals get deposited and absorbed by the solid fibers and within the pores. Due to this absorption of the FR chemicals in different phases of the fabrics, the FR chemicals did not wash out after multiple washings. As a result, after the TGA test the FR-treated fabrics possess a higher residual mass compared to the untreated fabrics.

### 3.4. Free Formaldehyde Content Detection

Given toxicological data and epidemiological findings gathered from occupational settings, in 2004 the International Agency for Research on Cancer (IARC) categorized formaldehyde as a Group 1 human carcinogen [53,54]. Allergy-induced contact dermatitis linked to clothing is one of the most debilitating skin conditions. Since 1926, the textile industry has employed resins containing formaldehyde to create fabrics with resistance to wrinkles [55]. According to previous data, the release of free formaldehyde in industrial practice in US textiles has been reduced from 2000 ppm to 100–200 ppm [56,57]. Although the product chosen for this observation was not specifically stated, it is reasonable to conclude that the textiles considered in this study might pertain to outerwear textiles.

**Figure 8:**
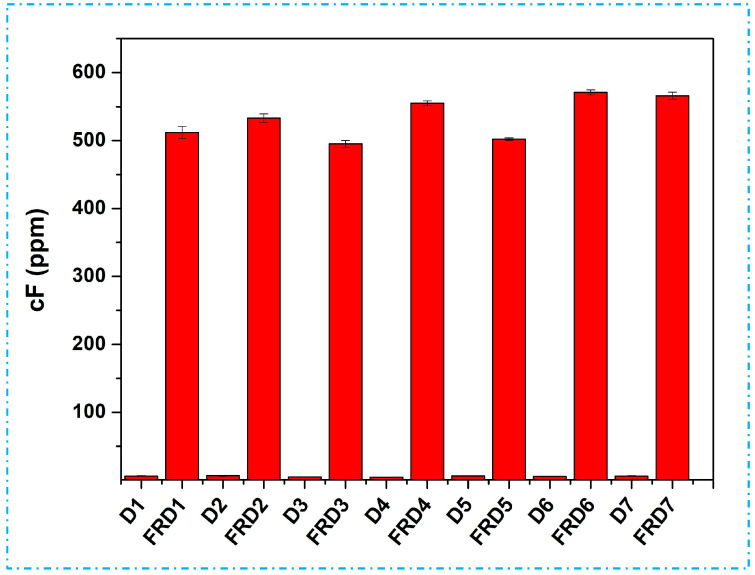
Free formaldehyde content (cF) of untreated (D1–D7) and FR-treated (FRD1–FRD7) jute fabrics.

In this research, subsequent to the FR treatment of the fabric samples, they were kept at room temperature for a period of one week. Following that, the least amount of free formaldehyde content (wF) was detected 495 ppm in FRD3 fabrics consecutive to 502 ppm (FRD5), 512 ppm (FRD1), 533 ppm (FRD2), 555 ppm (FRD4), 566 ppm (FRD7), and the maximum 571 ppm for FRD6 fabrics, respectively (Figure 8), and this might align with the FR chemical employed in this research. However, there was minimal formaldehyde content detected in the untreated samples, possibly attributed to external contamination. The main source of free formaldehyde was the crosslinking agent (KNITTEX CHN) and the FR chemical (ITOFLAM CPN), which supports earlier results of Drago Katovic. Cotton fabrics treated solely with an organophosphorus FR chemical without crosslinking were claimed to potentially possess free formaldehyde concentrations exceeding 300 ppm [58,59]. The results of this research do not comply with the standards for textiles intended for direct skin contact. According to Oeko-Tex Standard 100, for fabrics (like outerwear) and decorative materials that are not in direct contact with the skin, the permitted amount of free formaldehyde is 300 ppm, while it imposes a threshold of 75 ppm for fabrics in close proximity to the skin. Furthermore, the standard also stipulates a restriction for baby wear, requiring it to release less than 20 ppm of formaldehyde [60,61].

The development of environmentally friendly FR chemicals, as well as formaldehyde-free crosslinking and binding agents necessary to achieve effective flame-retardance functions, has received increased attention in recent research studies. Some studies have utilized formaldehyde-free FR treatments employing a non-methylol alternative for organophosphorus agents and incorporating plant-derived FR substances such as banana pseudostem sap [62], along with additives like branched polyethyleneimine (BPEI) and ammonium polyphosphate (APP) [63], phytic acid [64] and a synergistic flame retardant-containing silicon, phosphorus, and nitrogen [65].

## 4. Conclusions

This study aims to explore the potential for enhancing the flame-retardance properties of seven weave structures of jute fabrics by employing an organophosphorus FR chemical and to determine their efficacy. Substantial enhancements were attained in the thermal stability and FR capability of the jute fabrics. The FR-treated fabrics exhibited an improved potential to form chars, as observed when combustion thermal degradation occurred. The phosphorus and nitrogen element content in the SEM-EDS results indicate that the application of FR chemicals on the fiber surface took place during the treatment processes. The FTIR investigation demonstrated that the application of FR treatment did not impact the inherent properties and functional groups present on the surface of the fiber. The results from TGA indicated the onset of initial degradation in the FR-treated jute fabrics. Nonetheless, there was a considerable enhancement in residual mass, reaching nearly twice the value observed in the untreated fabrics for those treated with the flame retardant. After ten washes, the residual mass remains elevated compared to that of the jute fabrics without treatment, and this ultimately indicates the deposition of FR chemical on the jute fiber.

Among the fabric weaves used in this study, some differences in the FR performance were observed. Twill weaves resulted in a higher functionalization of FR and thermal degradation than the other types of weaves employed in the current study. On the contrary, the amount of formaldehyde found in the samples treated with FR surpasses the acceptable levels specified for textiles intended for direct skin contact. According to the specified results, although the residual mass of the TGA study between one weave and the other remained close, the initial and final degradation temperature for twill weaves (FRD4 and FRD5) was lower than the remaining weaves. The FRD6 fabric leads with the worst performance in free formaldehyde content. The employment of a melamine-based crosslinking agent in combination with the FR chemical is believed to result in an elevated level of free formaldehyde on the FR-treated fabrics. It can form links between the FR agent and cellulose; conversely, it makes the FR-treated fabric stiffer and promotes poor mechanical and hand properties. These substances influence the pyrolysis process, suppressing the generation of levoglucosan as well as combustible volatiles, fostering char formation, and thereby serving as flame retardants for cellulose [66]. To conclude, it can be said that no significant differences in thermal degradation and chemical properties were found between the weave designs used in this study. Therefore, such FR chemicals can be applied to exterior textiles other than clothing that is worn in contact with the skin, and for those applications where physical properties are not the prime concern but FR functions are.

This study advances the field of research on the application of natural fibers. Although jute fiber possesses a high production rate and strength, it has not been applied in many fields due to its limitations related to flammability. Our research will advance the field of natural fibers by enhancing its flame-retardancy. Therefore, these fiber-based fabrics could be used in many fields, including the field of protective textiles such as gloves. Although this study investigated the fundamentals of the flame-retardancy of our developed fabrics, this has not been investigated in consideration of the particular application of these newly developed fabrics. In the future, our research will investigate the application of these fabrics in high-heat hazards; hence, the feasibility of the application of these fabrics in the field of protective clothing would be explored.

## Figures and Tables

**Table 1:** Fabric specifications.

Sample ID	Weave Structure
D1	Warp Rib: 2/2
D2	Weft Rib: 2/2
D3	Basket weave (Matt): 4/4
D4	Twill 3/1
D5	Twill 2/2
D6	Plain 1/1
D7	4 ends Irregular Satin

**Table 2:** FR finish treatment process.

Chemicals	Commercial Name of the Chemicals	Amount in the Recipe (g/L)
FR chemical	ITOFLAM CPN	400
Crosslinking Agent	KNITTEX CHN	50
Catalyst	Phosphoric Acid (80%)	20
Pick-up %		75

**Table 3:** Color difference results of untreated and FR-treated fabrics.

Sample ID		Color Difference (ΔE)
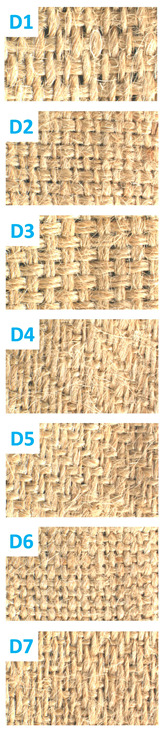	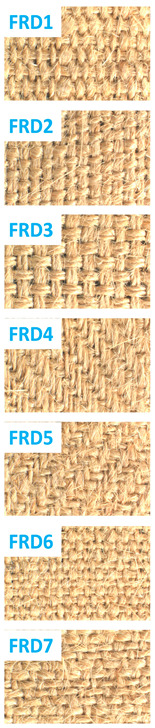	6.16
		6.28
		6.28
		4.24
		3.92
		5.35
		5.90

## Data Availability

The original contributions presented in the study are included in the article, further inquiries can be directed to the corresponding author.
